# Role of mental health and quality of life in adherence and effectiveness of a motivational exercise program to improve weight and functionality: “The way to change diabetes”.

**DOI:** 10.1192/j.eurpsy.2024.1023

**Published:** 2024-08-27

**Authors:** J. M. Pelayo-Terán, S. Vega-García, Z. Gutiérrez-Hervás, M. E. García-Llamas, A. Díez-Hernández, M. E. López Crespo, D. J. Durán Román, H. J. Bilo, Y. Zapico Merayo

**Affiliations:** ^1^Psiquiatría y Salud Mental; ^2^Unidad de Calidad y Seguridad del Paciente, Hospital El Bierzo. Gerencia de Asistencia Sanitaria del Bierzo (GASBI). Gerencia Regional de Salud (SACYL), Ponferrada; ^3^Área de Medicina Preventiva y Salud Pública. Departamento de Ciencias Biomédicas, Universidad of León, León; ^4^Grupo CB07/09/2001, Centro de Investigacion Biomédica en Red de Salud Mental (CIBERSAM), Madrid; ^5^Hospital El Bierzo. Gerencia de Asistencia Sanitaria del Bierzo (GASBI). Gerencia Regional de Salud (SACYL), Ponferrada, Spain; ^6^Servicio de Endocrinología, Hospital El Bierzo. Gerencia de Asistencia Sanitaria del Bierzo (GASBI). Gerencia Regional de Salud (SACYL), Ponferrada, Spain; ^7^Bas van de Goor Foundation, Arnhem, Netherlands; ^8^Delegación Ponferrada, Asociación Salud Mental León, Ponferrada, Spain; ^9^University Medical Center Groningen, Department of Internal Medicine, University of Groningen, Groningen; ^10^Isala Diabetes Research Center, Zwolle, Netherlands

## Abstract

**Introduction:**

Exercise and other lifestyles are key treatment strategies to improve diabetes outcome, prevent cardiovascular risk and may also result in further results in quality if life and emotional symptoms.

**Objectives:**

To evaluate the effectiveness of an exercise intervention program for people with diabetes or cardiovascular risk.

To evaluate the influence of previous metal health and quality of life status in the results.

**Methods:**

61 people with a type 2 diabetes or cardiovascular risk factors were recruited from health primary health centers in Ponferrada (EL Bierzo), including patients from the mental health association. After informed consent they were included in a 20 week, twice a week supervised walking training program to improve exercise and other lifestyles. A poster used for advertisement of the adtivity (“the way/walk to change diabetes”) is displayed in image 1). Baseline and after 20 weeks BMI and Waist perimeter were assessed, quality of life was evaluated with EQ-5D-5L and WHO-5 scales and the weekly steps walked were recorded previously and after the intervention with the subject usual mobile device.

Differences in the variables were compared with Paired Ts and repeated ANCOVAs measures adjusted by gender, age and initial steps.

**Results:**

46 subjects (75.4%) completed more than 90% of the sessions and 3 more 70-90%. The 19.7% that did not complete had worse scores in SF-12 Role Physical (t 2.261, p=0.041) and Role Emotional (t:2.048, p=0.045) and Mental Component Summary (t:2,313; p=0,036) and WHO5 Total Score (t:2.101; p=0,040) at Baseline. Main reasons for dropout (Image 2) were health related problems (50%) and adherence to exercise and motivation problems (31.25%).

Those who completed the training improve number of weekly steps (baseline: 42022,92 +- 18836,35, final: 66448.06 +-28914,58; t:5.038; p<0.001), BMI (29.45 +-4.66 to 28.25 +-4.09 kg/m2; t:5.629; p<0.001), waist (from 107,34 + 9.98 to 102,88 +9,79 cm; t:6,840; p<0.001) and the EoQ-5D-EL VAS (form 72.88 to 82.42; t:6.122; p<0.001, image 3). The increase in the steps correlated directly with the improvement in the EoQ VAS (r:0.308; p=0.033).

**Image:**

**Figure:**
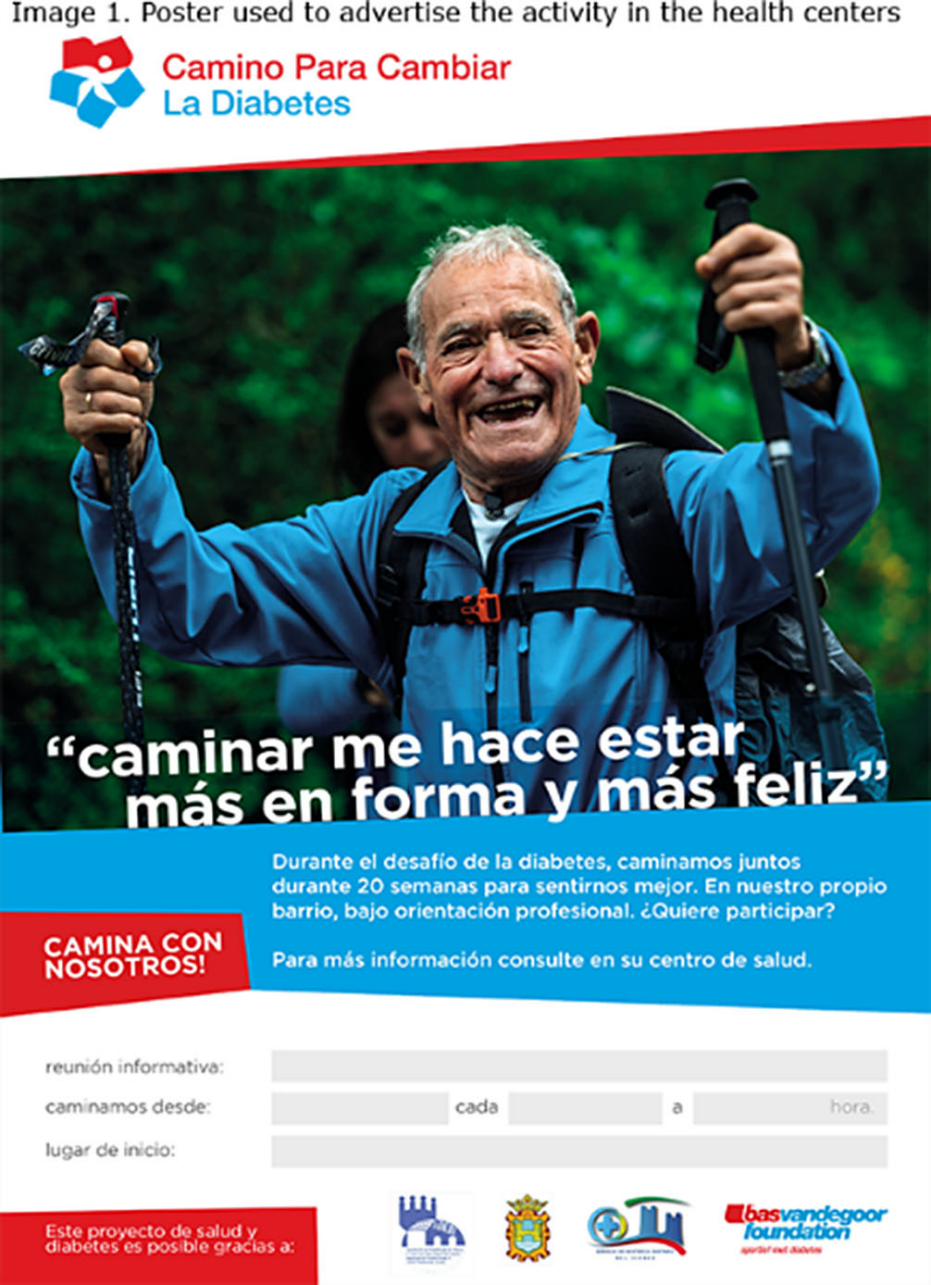

**Image 2:**

**Figure:**
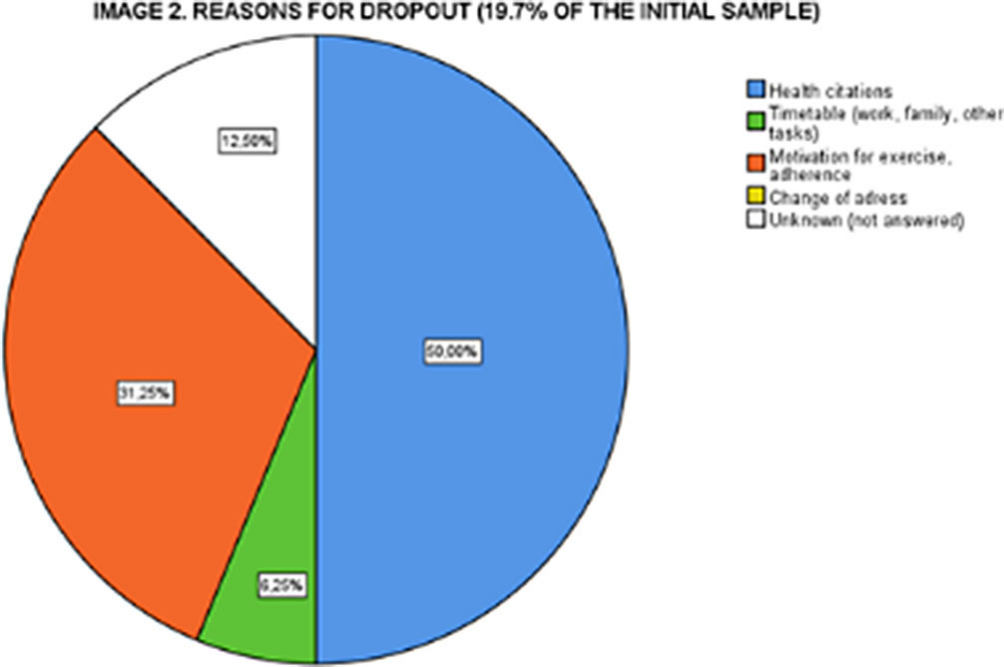

**Image 3:**

**Figure:**
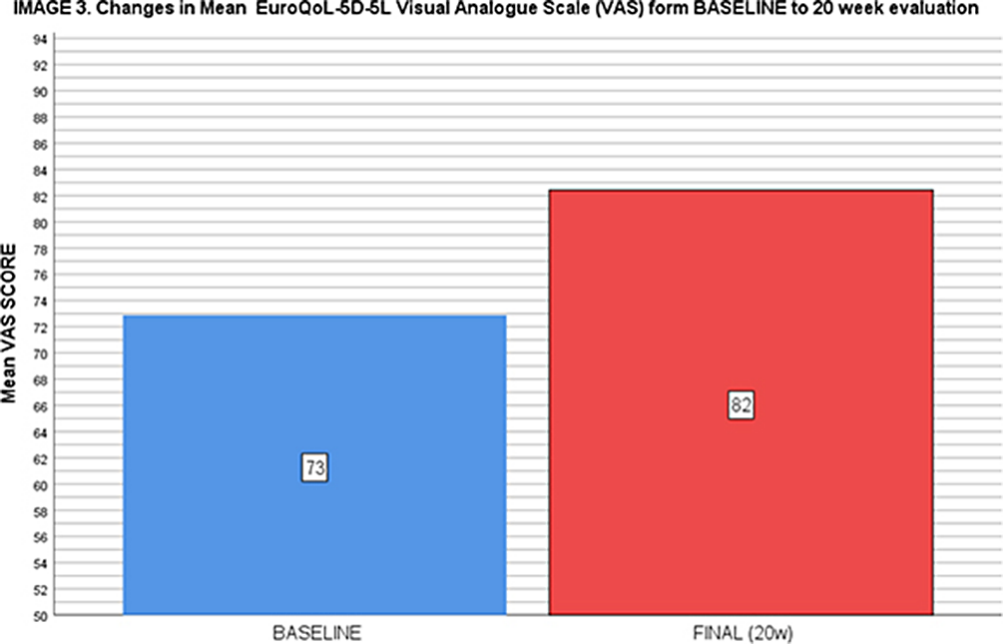

**Conclusions:**

Exercise and lifestyle supervised intervention programs appear to be useful to improve physical health, wellbeing, emotional symptoms and quality of life in people with diabetes and cardiovascular risk.

Factors associated to higher dropout rates were previous limited quality of life scores and mental health worse status. These could be related with limited motivation and adherence to the program and may be of interest to develop specific strategies for these high-risk groups.

Studies focused on the long-term effect of the program are warranted.

**Disclosure of Interest:**

None Declared

